# Role of cytokine levels in pathogen classification and prognosis of pediatric septic shock

**DOI:** 10.3389/fimmu.2026.1714948

**Published:** 2026-01-27

**Authors:** Guangyan Du, Haixin Huang, Ruichen Zhang, Chengjun Liu, Yueqiang Fu

**Affiliations:** 1 Department of Critical Care Medicine, Children’s Hospital of Chongqing Medical University, Chongqing, China; 2 Children’s Hospital of Chongqing Medical University National Clinical Research Center for Child Health and Disorders, Ministry of Education, Key Laboratory of Child Development and Disorders, Chongqing, China; 3 Children’s Hospital of Chongqing Medical University, Chongqing Key Laboratory of Child Rare Diseases in Infection and Immunity, Chongqing, China; 4 Department of Pediatric Pulmonology, Sichuan University West China Second University Hospital, Chengdu, China

**Keywords:** children, IL-10, IL-6, mortality, septic shock

## Abstract

**Background:**

The pathogenic role of inflammatory cytokine levels in children with septic shock has not been completely clarified. The aim of this study was to investigate the relationships between the early concentrations of inflammatory cytokines, pathogen classification, and 28-day mortality in children with septic shock.

**Method:**

We retrospectively analyzed the early expression of cytokines in children admitted to the pediatric intensive care unit (PICU) of a tertiary pediatric hospital due to septic shock between July 2019 and September 2024.

**Results:**

A total of 189 children with septic shock were included and 68 died within 28 days of hospitalization. The plasma levels of IL-6 (P = 0.001), IL-10 (P < 0.001), IFN-γ (P = 0.002), and TNF-α (P = 0.014) were significantly higher in the nonsurvivor group than in the survivor group. Multivariate Cox regression analysis revealed that the IL-6 level was an independent risk factor for 28-day mortality after controlling platelet count and lactate, lactate dehydrogenase, Hb, IFN-γ, and TNF-α levels. ROC analysis revealed the AUC values of the IL-6, IL-10, IFN-γ, and TNF-α levels were 0.64, 0.68, 0.64 and 0.61, respectively, and that the optimal cutoff values were 414.92 pg/ml, 29.66 pg/ml, 1.605 pg/ml and 0.725 pg/ml, respectively. According to these cutoff values, the survival curves of the groups with high levels of IL-6, IL-10, IFN-γ, and TNF-α differed significantly from those with low levels (p < 0.001, p < 0.001, p = 0.001, and p = 0.001, respectively). In children with positive fluid cultures, there was no statistically significant difference in cytokines levels between the gram-positive bacterial (G+) and gram-negative bacterial (G-) infection groups. However, in children with positive blood cultures, the levels of IL-6 (p = 0.005) and IL-10 (p = 0.003) were significantly higher in the G- group than in the G+ group.

**Conclusion:**

Elevated IL-6 and IL-10 levels are valuable for predicting 28-day mortality and identifying gram-negative bacteremia in pediatric patients with septic shock. IFN-γ and TNF-α levels also have significant value for predicting 28-day mortality. Moreover, the IL-6 level was an independent risk factor for 28-day mortality.

## Introduction

Sepsis is the leading cause of death among patients in the intensive care unit (ICU), accounting for 19.7% of deaths worldwide (1). There were an estimated 2·9 million sepsis-related deaths worldwide among children younger than 5 years in 2017 (1). Septic shock is a serious complication of sepsis and an important risk factor for mortality. The prevalence of septic shock among children with sepsis was reported to be 53.7% in resource-rich settings and 81.3% in resource-poor settings, and the mortality rates were 10.8% and 33.5%, respectively (2).

Sepsis is a severe clinical syndrome caused by an inappropriate host response to infection (3). Cytokines are soluble low-molecular-weight proteins secreted by immune cells (4). Pathogen-associated molecular patterns (PAMPs) interact with host immune cells, resulting in the release of large amounts of cytokines, which can lead to organ dysfunction when cytokine levels exceed a certain threshold (5). In adult studies, the levels of IL-6 and IL-10 have been used to differentiate between sepsis and nonsepsis and between sepsis and septic shock, and have been shown to influence 28-day mortality in septic patients (6); they also can be used to predict Gram staining results for pathogens of bloodstream infections (7). In pediatric studies, IL-6 and IL-10 have been associated with early childhood sepsis (8, 9), and their elevation was closely associated with the development of septic shock in cancer patients (10), including an ability to differentiate between Gram stains for bacteremic pathogens (11). Furthermore, to some extent, the IL-6 level has been shown to differentiate sepsis from noninfectious systemic inflammatory response syndrome (SIRS) (12) and to be a weak predictor of mortality in patients with septic shock (13). *In vitro* cytokine absorption has been shown to reduce the demand for postoperative inotropes, and the SOFA score and APACHE II score predicted in-hospital mortality in patients with acute kidney injury and septic shock after cardiac surgery (14).

Markedly elevated cytokine levels may be important factors in the progression of septic shock, but studies on cytokine levels in children with septic shock are lacking. The purpose of this study was to investigate the relationships between the early expression of inflammatory cytokine (IL-2, IL-4, IL-6, IL-10, IL-17A, IFN-γ, and TNF-α), pathogen classification, and 28-day mortality in children with septic shock. The ability of cytokine levels to predict mortality was also evaluated using receiver operating characteristic curves (ROCs).

## Methods

### Study design

This retrospective cohort study analyzed the clinical data and outcomes of children with septic shock who were admitted to the Pediatric Intensive Care Unit (PICU) at the Children’s Hospital of Chongqing Medical University between July 2019 and September 2024. Patient data were anonymized prior to analysis, and the study was approved by the Institutional Review Committee at the Children’s Hospital of Chongqing Medical University. Due to the retrospective nature of this study, a waiver of informed consent was granted.

Patients were identified on the basis of discharge diagnostic data and laboratory parameters in the electronic health database. The inclusion criteria were children who were admitted to the PICU for septic shock and who underwent plasma cytokine level testing within 24 hours of admission to the PICU. Septic shock was diagnosed as significant cardiovascular dysfunction due to infection according to the 2017 Clinical Practice Guidelines (15), the 2020 Pediatric Guidelines (3), and the 2024 International Consensus (2). The exclusion criteria included the following: 1) Children with septic shock in PICU who were not tested for cytokines levels. 2) Children with septic shock who underwent cytokine level testing in other departments. 3) Children who were transferred to the PICU after surgery solely for postoperative ventilation support rather than antishock treatment, and were transferred back to the surgical ward the next day.

### Data collection and definition of variables

The following demographic and laboratory data were collected: age; weight; sex; comorbidities; white blood cell (WBC) and platelet (PLT) counts; hemoglobin (Hb), procalcitonin (PCT), C-reactive protein (CRP), lactate, lactate dehydrogenase (LDH); and plasma cytokine (IL-2, IL-4, IL-6, IL-10, IL-17A, IFN-γ, and TNF-α) levels, as well as culture results of fluid from suspected sites of infection.

Additionally, disease severity scores were calculated, including the Pediatric Index of Mortality-3 (PIM-3) (16), the Pediatric Risk of Mortality-III (PRISM-III) score (17), and the Pediatric Sequential Organ Failure Assessment (pSOFA) score (18). Furthermore, the requirements for intravenous immune globulin (IVIG) and continuous renal replacement therapy (CRRT) were also recorded. The primary outcome was 28-day mortality. All of the above data were obtained from the electronic medical records and manually reviewed.

### Statistical analysis

Statistical analysis was performed using R 4.3.0 and R 4.5.2 software. Continuous variables are expressed as medians (interquartile ranges), and categorical variables are expressed as frequencies (percentages). The rank-sum test was used for two independent samples and for multiple groups of samples, whereas the chi-square test or Fisher’s exact test was used for categorical variables. Multivariate Cox proportional hazards model analysis was used to assess the correlation with 28-day mortality. A receiver operating characteristic (ROC) curve was used to evaluate the diagnostic performance of the cytokine-level indicators for mortality. The area under the curve (AUC) and the optimal cutoff value were subsequently calculated. Kaplan–Meier curve analysis and the log-rank test were performed according to the optimal cutoff values for cytokines levels to evaluate the 28-day survival rate. A p value of less than 0.05 was considered statistically significant.

## Results

### Demographic data and baseline characteristics

A total of 189 children with septic shock were included in the study. Among those included, 68 (35.98%) died within 28 days of hospitalization. The clinical features of the children are presented in **Table 1**. The table shows that there were no statistically significant differences in sex, weight, or age between the two groups (p > 0.05). In terms of laboratory tests, WBC count, Hb level, and PLT count were significantly lower in the nonsurvivors. LDH, IL-6, IL-10, IFN-γ, and IFN-α levels were significantly higher in the nonsurvivors. CRP and PCT levels were lower in the nonsurvivors, although the differences between the two groups were not statistically significant.

**Table 1:** Clinical features of children with septic shock and laboratory parameters measured at the time of admission to the PICU.

Variables	Total (n = 189)	Survivors (n = 121)	Nonsurvivors (n = 68)	p
Sex(male), n (%)	105 (55.56)	70 (57.85)	35 (51.47)	0.397
Weight(kg), M (IQR)	18.00 (10.50, 35.00)	18.00 (10.50, 35.00)	18.00 (11.38, 33.50)	0.992
Age (month), M (IQR)	68.00 (18.00, 138.00)	71.00 (20.00, 139.00)	63.50 (15.00, 135.75)	0.635
WBC(x10^9^/l), M (IQR)	6.04 (2.08, 12.68)	7.23 (3.04, 13.32)	4.39 (0.76, 12.11)	0.012
Hb (g/l), M (IQR)	95.00 (78.00, 114.00)	99.00 (86.00, 116.00)	83.00 (70.75, 109.00)	0.002
PLT(x10^9^/l), M (IQR)	127.00 (45.00, 240.00)	146.00 (67.00, 253.00)	67.50 (27.75, 185.00)	<0.001
CRP(mg/l), M (IQR)	68.82 (21.69, 121.67)	71.00 (20.38, 120.54)	62.66 (22.67, 123.19)	0.530
PCT(ng/ml), M (IQR)	27.90 (3.54, 100.00)	28.82 (2.33, 100.00)	25.48 (7.20, 88.69)	0.588
Lactate(mmol/L), M (IQR)	1.70 (1.10, 3.60)	1.40 (1.00, 2.50)	2.60 (1.67, 4.93)	<0.001
LDH(U/L), M (IQR)	487.00 (290.00, 899.00)	440.00 (259.00, 678.00)	660.00 (341.52, 1676.25)	<0.001
IL-2(pg/ml), M (IQR)	0.72 (0.01, 2.04)	0.62 (0.01, 2.50)	0.83 (0.34, 1.66)	0.666
IL-4(pg/ml), M (IQR)	0.49 (0.01, 1.38)	0.32 (0.01, 1.37)	0.73 (0.01, 1.42)	0.472
IL-6(pg/ml), M (IQR)	813.41 (71.28, 7016.10)	348.12 (43.61, 3324.36)	2241.93 (283.64, 14087.71)	0.001
IL-10(pg/ml), M (IQR)	63.99 (13.45, 393.40)	36.67 (9.14, 220.76)	185.91 (38.14, 952.87)	<0.001
IL-17A(pg/ml), M (IQR)	2.50 (0.01, 7.31)	1.54 (0.01, 7.68)	3.78 (0.87, 6.90)	0.078
IFN-γ(pg/ml), M (IQR)	1.61 (0.37, 9.53)	1.28 (0.23, 5.13)	3.22 (0.62, 23.97)	0.002
TNF-α(pg/ml), M (IQR)	1.19 (0.39, 3.39)	0.90 (0.17, 2.50)	1.60 (0.79, 3.74)	0.014
PRISM-III score, M (IQR)	12.00 (8.00, 16.00)	11.00 (7.00, 14.00)	15.00 (11.00, 19.00)	<0.001
PIM-3, M (IQR)	0.02 (0.01, 0.05)	0.02 (0.01, 0.04)	0.03 (0.02, 0.07)	0.005
pSOFA score, M (IQR)	6.00 (4.00, 9.00)	6.00 (4.00, 9.00)	7.50 (5.00, 11.00)	0.002
Comorbidity				
Hematologic malignancy, n(%)	34 (17.99)	16 (13.22)	18 (26.47)	0.023
Tumor, n (%)	37 (19.58)	21 (17.36)	16 (23.53)	0.305
Rheumatism, n (%)	21 (11.11)	13 (10.74)	8 (11.76)	0.83
IVIG therapy, n (%)	115 (60.85)	80 (66.12)	35 (51.47)	0.048
CRRT, n (%)	94 (49.74)	47 (38.84)	47 (69.12)	<0.001

IQR interquartile range; M, median; CRP, C-reactive protein; CRRT, continuous renal replacement therapy; Hb, hemoglobin; IFN-γ, interferon-γ; IL-2, interleukin-2; IL-4, interleukin-4; IL-6, interleukin-6; IL-10, interleukin-10; IL-17A, interleukin 17A; IVIG, intravenous immune globulin; LDH, lactate dehydrogenase; PCT, procalcitonin; PLT, platelet; PIM-3, International Society of Pediatric Index of Mortality-3; PRISM-III, Pediatric Risk of Mortality-III; pSOFA, Pediatric Sequential Organ Failure Assessment; TNF-α, tumor necrosis factor-alpha; WBC, white blood cell.

In terms of comorbidities, the proportions of patients with rheumatic diseases (13% vs. 8%, p = 0.83) and tumors (21% vs. 16%, p = 0.305) were similar between the two groups, while the proportion of patients with hematologic malignancies (16% vs. 18%, p = 0.023) was significantly greater among nonsurvivors (**Table 1**). Moreover, PRISM-III (11 vs. 15, p < 0.001), PIM-3 (0.02 vs. 0.03, p = 0.005), and pSOFA (6 vs. 7.5, p = 0.002) scores were significantly higher in the nonsurvivor group than in the survivor group.

In addition, the proportion of patients treated with IVIG was higher in the survivor group (66.12% vs. 51.47%, p = 0.048). The percentage of nonsurvivors who needed CRRT was significantly higher than that of survivors (38.84% vs. 69.12%, p < 0.001) (**Table 1**).

The differences in cytokine levels between the survivors and nonsurvivors at different ages were explored. Among children < 5 years, compared with those in the survivors, the IL-6 and IFN-γ levels in the nonsurvivors were significantly elevated. Among children ≥ 5 years, the nonsurvivors had significantly higher levels of IL-6, IL-10, IL-17A, IFN-γ, and TNF-α than the survivors did (**Supplementary Table 1**).

The differences in cytokine levels between the survivors and nonsurvivors with different pSOFA scores were also investigated. Among patients with pSOFA scores ≤ 5, the nonsurvivors had significantly higher IL-10 levels than the survivors. Among patients with pSOFA scores > 5, IL-6, IL-10, and IFN-γ levels were significantly elevated in nonsurvivors compared with survivors (**Supplementary Table 2**).

### Cox regression analysis of the predictors of 28-day mortality

IL-6, IL-10, IFN-γ, and TNF-α levels and other significant blood indicators were analyzed using multivariable Cox proportional hazards models, and the results are presented in **Table 2**. Univariate Cox analysis indicated that PLT count and lactate, LDH, IL-6, IFN-γ, TNF-α, and Hb levels were associated with the 28-day mortality rate (p < 0.05). Parameters with significant results in univariate analysis were evaluated using multivariate Cox regression analysis. Lactate (p < 0.001), LDH (p < 0.001), and IL-6 (p = 0.006) levels were independently associated with the 28-day mortality rate of patients in the multivariate analysis.

**Table 2:** Regression analysis of important blood indicators.

Variables	Univariable HR (95% CI)	p	Multivariable HR (95% CI)	p
PLT, M (IQR)	0.99 (0.99~0.99)	0.002	1.00 (1.00~1.00)	0.061
Lactate, M (IQR)	1.15 (1.10~1.21)	<0.001	1.14 (1.07~1.21)	<0.001
LDH, M (IQR)	1.01 (1.01~1.01)	<0.001	1.01 (1.01~1.01)	0.024
IL-6, M (IQR)	1.01 (1.01~1.01)	0.006	1.01 (1.01~1.01)	0.008
IL-10, M (IQR)	1.00 (1.00~1.00)	0.088	1.00 (1.00~1.00)	0.864
IFN-γ, M (IQR)	1.01 (1.01~1.01)	0.005	1.00 (1.00~1.00)	0.451
TNF-α, M (IQR)	1.01 (1.01~1.01)	0.002	1.00 (1.00~1.01)	0.073
Hb, M (IQR)	0.99 (0.98~0.99)	0.024	1.00 (0.99~1.01)	0.890
WBC, M (IQR)	0.99 (0.96~1.01)	0.301	1.01 (0.99~1.04)	0.290

IQR, interquartile range; M, median; CI, confidence Interval; Hb, hemoglobin; HR, hazards Ratio; IFN-γ, interferon-γ; IL-2, interleukin-2; IL-4, interleukin-4; IL-6, interleukin-6; IL-10, interleukin-10; IL-17A, interleukin 17A; LDH, lactate dehydrogenase; PLT, platelet; TNF-α, tumor necrosis factor-alpha; WBC, white blood cell.

### AUC values for cytokine parameters for 28-day mortality determined by ROC analysis

**Figure 1:**
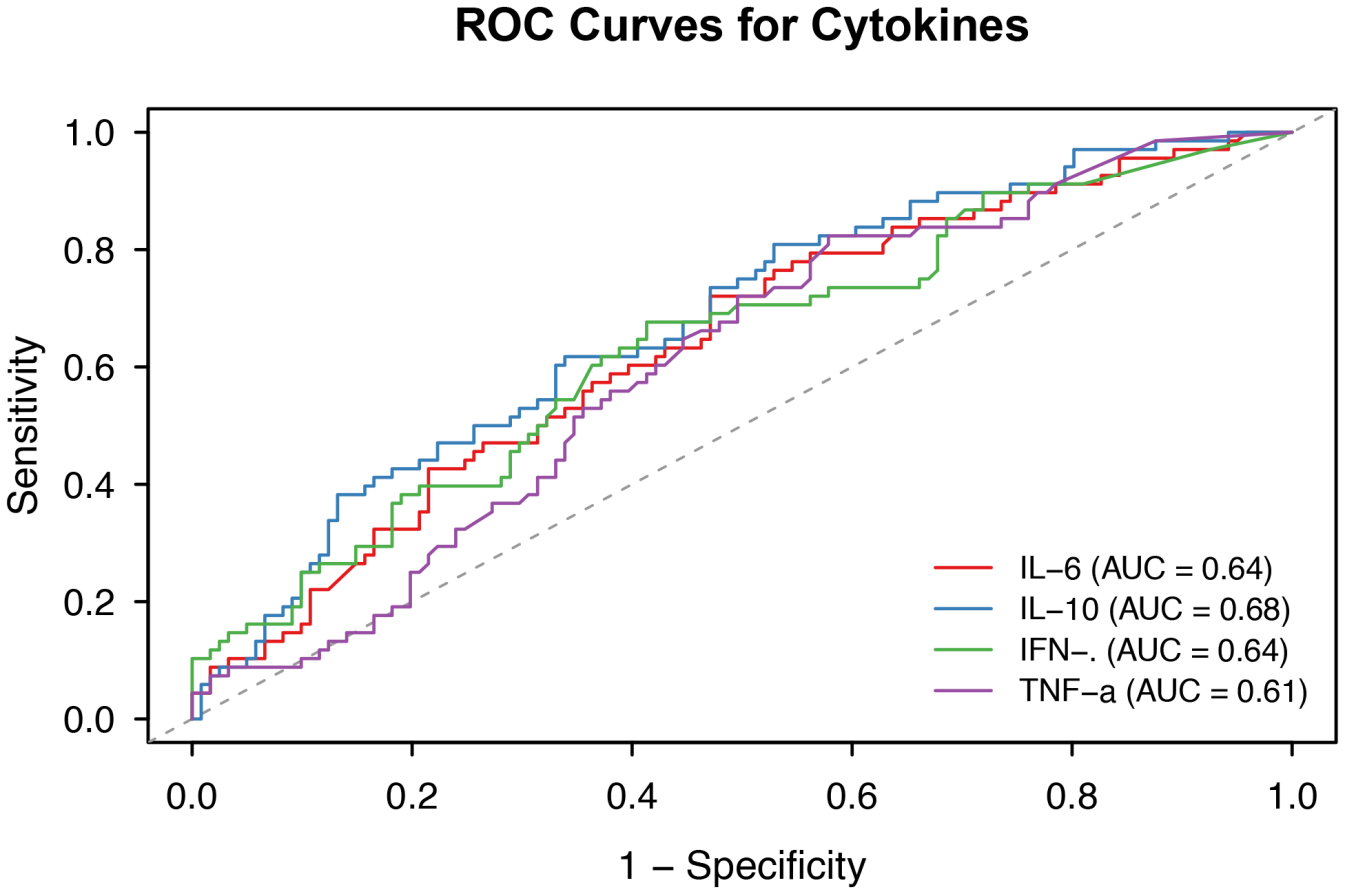
ROC curve analyses of 28-day mortality in children with septic shock. IL-6, IL-8, IFN-γ and TNF-α levels predict mortality. AUC, area under the curve; IL-6, interleukin-6; IL-10, interleukin-10; IFN-γ, interferon-γ; TNFα, tumor necrosis factor-alpha; ROC, receiver operating characteristic.

The area under the receiver operating characteristic curve (AUC) was used to predict mortality. As shown in **Table 3**, the AUC of IL-6 for predicting 28-day mortality was 0.64 (95% confidence interval [CI], 0.56–0.72; p = 0.001), that of IL-10 was 0.68 (95% CI, 0.60–0.76; p < 0.001), that of IFN-γ was 0.64 (95% CI, 0.55–0.72; p = 0.001), and that of TNF-α was 0.61 for (95% CI, 0.53–0.69; p = 0.006) (**Table 2**, **Figure 1**). The optimal cutoff values for 28-day mortality in children with septic shock were 414.92 pg/mL for IL-6, 29.66 pg/mL for IL-10, 1.605 pg/mL for IFN-γ and 0.725 pg/mL for TNF-α. The combination of the aforementioned cytokines, including IL-6 + IL-10 (AUC = 0.68) and IL-6 + IFN-γ (AUC = 0.68), did not improve diagnostic performance (**Supplementary Figure 1**).

**Table 3:** Cutoff and AUC values for cytokine parameters for 28-day mortality.

Parameter	AUC (95% CI)	Cut off	Specificity (95% CI)	Sensitivity (95% CI)	Accuracy (95% CI)	PPV (95% CI)	NPV (95% CI)	p
IL6 (pg/ml)	0.64 (0.56-0.72)	414.92	0.72 (0.61 - 0.83)	0.53 (0.44 - 0.62)	0.60 (0.52-0.67)	0.77 (0.68 - 0.86)	0.46 (0.37 - 0.56)	0.001
IL10 (pg/ml)	0.68 (0.60-0.76)	29.66	0.81 (0.72 - 0.90)	0.47 (0.38 - 0.56)	0.59 (0.52-0.66)	0.81 (0.72 - 0.91)	0.46 (0.37 - 0.55)	<0.001
IFN-γ (pg/ml)	0.64 (0.55-0.72)	1.605	0.68 (0.57 - 0.79)	0.59 (0.50 - 0.67)	0.62 (0.55-0.69)	0.76 (0.68 - 0.85)	0.48 (0.38 - 0.58)	0.001
TNFα (pg/ml)	0.61 (0.53-0.69)	0.725	0.82 (0.73 - 0.91)	0.42 (0.33 - 0.51)	0.57 (0.49-0.64)	0.81 (0.71 - 0.91)	0.44 (0.36 - 0.53)	0.006

AUC, area under the receiver operating characteristic curve; IL-6, interleukin-6; IL-10, interleukin-10; IFN-γ, interferon-γ; TNFα, tumor necrosis factor-alpha; PPV, positive predictive value; NPV, negative predictive value.

### Stratified analysis

**Figure 2:**
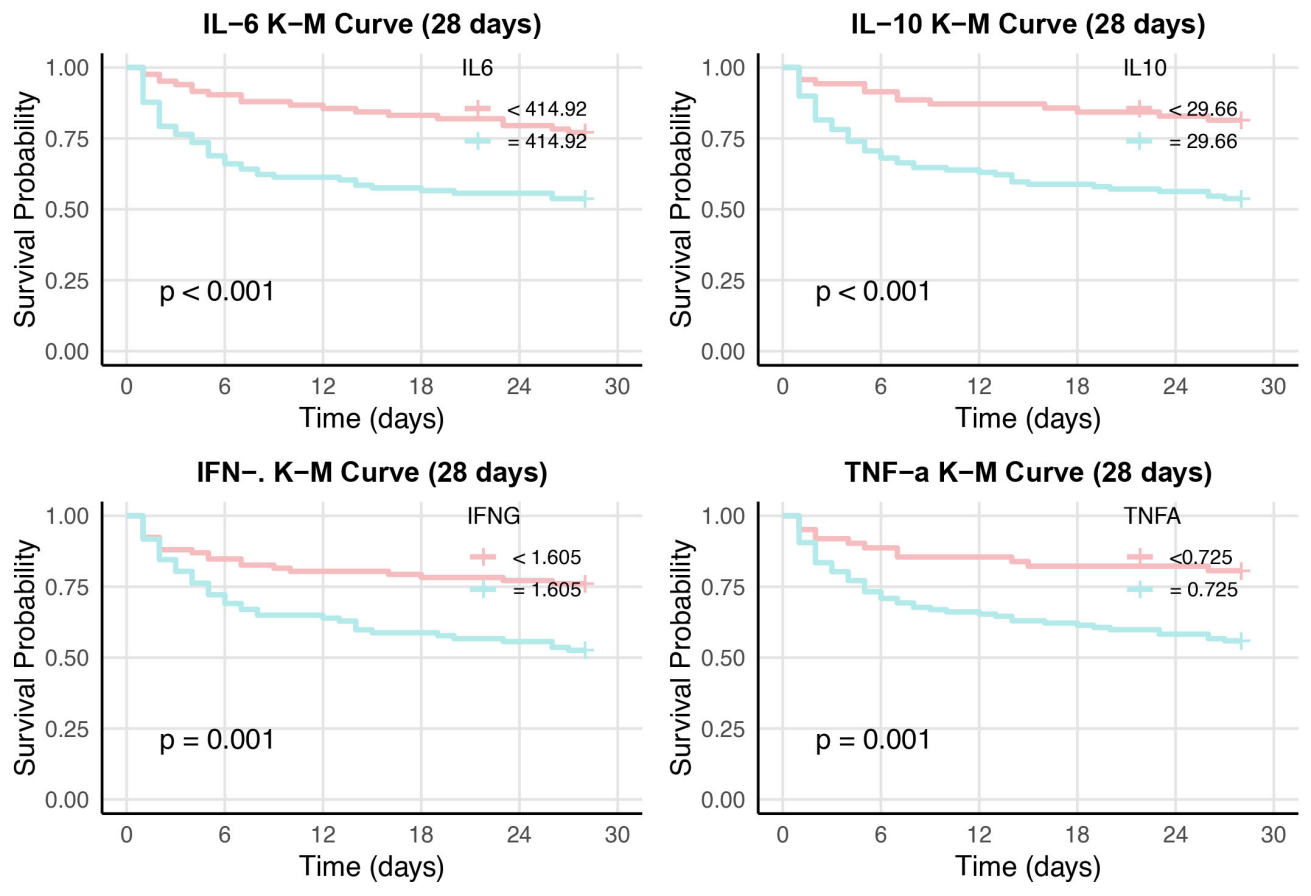
Kaplan-Meier survival curves between children with high and low cytokine (IL-6, IL-10, IFN-γ and TNF-α, respectively). The survival curves censored to 28 days.

Kaplan–Meier curve analysis and log-rank test analysis were performed according to the optimal cutoff value for 28-day mortality, and the high-IL-6 (≥ 414.92 pg/mL) and low-IL-6 (< 414.92 pg/mL) group, the high-IL-10 (≥ 29.66 pg/mL) and low-IL-10 (< 29.66 pg/mL) group, the high-IFN-γ (≥ 1.605 pg/mL) and low-IFN-γ (< 1.605 pg/mL) group, and the high-TNF-α (≥ 0. 0.725 pg/mL) and low TNF-α (< 0.725 pg/mL) group 28-day survival curves (**Figure 2**) were compared. According to the log-rank test, the survival curves of the high-IL-6, IL-10, IFN-γ, and TNF-α groups significantly differed from those of the low-IL-6, IL-10, IFN-γ, and TNF-α groups (p < 0.001, p < 0.001, p = 0.001, p = 0.001, respectively) (**Figure 2**).

### Characteristics of the pathogenic bacteria

A total of 144 bacterial strains, including fungi, were detected in all positive body fluid cultures. As shown in **Table 4**, gram-negative bacteria were the main pathogens (50.69%), of which *Klebsiella* was the most common (20.55%). However, overall, the most common bacterial infection was caused by *Staphylococcus*.

**Table 4:** Frequencies of bacteria in the positive cultures.

Variables	Frequency (%)	Blood	Sputum	Urine	Etc^a^
Gram-positive, n (%)	58(40.28%)				
*Staphylococcus* species	35(24.3% )	14	14		7
*Streptococcus* species	13(9.0%)	7	5		1
Others	10(6.9%)	4	1	3	2
Gram-negative, n (%)	73(50.69%)				
*Klebsiella* species	15(10.4%)	6	7		2
*Pseudomonas aeruginosa*	12(8.3% )	4	7		1
*Escherichia coli*	14(9.7%)	8	3		3
*Acinetobacter baumannii*	8(5.6%)		7		2
Others	24(16.7%)	7	14	1	2
Fungus, n (%)	13(9.0%)				
*Candida*	13(9.0% )		10	1	2

### Characteristics of clinical indicators of fluid-cultured bacteria

The impact of the Gram classification of blood culture-positive bacteria on cytokine expression was then investigated (**Table 5**). According to the culture results, the children were divided into gram-positive (G+) and gram-negative (G-) groups. Owing to the small number of children coinfected with G+ and G- bacteria (n = 4), they were excluded from the statistical analysis. Among the 189 children with septic shock, 40 had positive blood cultures, including 18 (45.00%) in the G+ group and 22 (55.00%) in the G- group.

**Table 5:** Laboratory parameter results derived from blood culture results.

Variables	Total (n = 40)	G+ group (n = 18)	G- group(n = 22)	p
WBC(x10^9^/l), M (IQR)	2.60 (0.54, 9.10)	3.95 (2.15,11.34)	1.23 (0.13,4.15)	0.031
CRP(mg/l), M (IQR)	79.25 (44.40, 144.67)	79.50 (26.17,139.98)	79.25 (59.73,141.50)	0.587
PCT(ng/ml), M (IQR)	38.40 (5.65, 100.00)	38.40 (4.00,99.08)	36.33 (6.80,100.00)	0.611
Lactate(mmol/L), M (IQR)	2.20 (1.20, 3.60)	1.45 (1.02,2.50)	2.40 (1.77,4.58)	0.035
LDH(U/L), M (IQR)	399.80 (288.50, 551.25)	460.50 (293.50,575.75)	346.50 (289.25,495.18)	0.338
IL-2(pg/ml), M (IQR)	1.10 (0.27, 1.79)	0.80 (0.14,1.64)	1.29 (0.35,2.30)	0.530
IL-4(pg/ml), M (IQR)	0.99 (0.01, 1.88)	0.82 (0.01,1.75)	1.29 (0.50,1.86)	0.548
IL-6(pg/ml), M (IQR)	3560.90 (347.71, 14087.71)	1669.63 (179.19,2857.24)	10063.81 (1779.62,17475.90)	0.005
IL-10(pg/ml), M (IQR)	108.56 (47.68, 770.96)	56.92 (35.49,84.75)	696.43 (113.27,1226.29)	0.003
IL-17A(pg/ml), M (IQR)	3.60 (0.85, 7.14)	1.10 (0.01,6.74)	5.31 (2.25,7.26)	0.131
IFN-γ(pg/ml), M (IQR)	2.75 (1.19, 8.50)	3.27 (1.06,12.57)	2.50 (1.32,4.16)	0.532
TNF-α(pg/ml), M (IQR)	1.63 (0.74, 3.78)	1.73 (0.70,7.58)	1.54 (1.03,3.43)	1
PRISM-III, M (IQR)	13.50 (8.50, 16.25)	12.00 (4.00,15.50)	13.50 (11.00,17.50)	0.134
PIM-3, M (IQR)	0.03 (0.01, 0.04)	0.03 (0.01,0.04)	0.03 (0.02,0.05)	0.609
pSOFA,M (IQR)	6.00 (4.00, 9.00)	4.00 (3.00,8.25)	6.50 (5.25,8.75)	0.053

IQR, interquartile range; M, median; CRP, C-reactive protein; G-, gram-negative; G+, gram-positive; IFN-γ, interferon-γ; IL-2, interleukin-2; IL-4, interleukin-4; IL-6, interleukin-6; IL-10, interleukin-10; IL-17A, interleukin 17A; LDH, lactate dehydrogenase; PCT, procalcitonin; PIM-3, International Society of Pediatric Index of Mortality-3; PRISM-III, Pediatric Risk of Mortality-III; pSOFA, Pediatric Sequential Organ Failure Assessment; TNF-α, tumor necrosis factor-alpha; WBC, white blood cell.

As shown in **Table 5**, the WBC counts (3.95 vs. 1.23 × 10^9^/l, P = 0.031) was significantly higher in Group G +. In contrast, the levels of IL-6 (1669.63 vs. 10063.81 pg/mL, p = 0.005), IL-10 (56.92 vs. 696.43 pg/mL, p = 0.003), and lactate (1.45 vs. 2.40 mmol/L, p = 0.035) were significantly higher in Group G-. No statistically significant differences were observed in CRP level, PCT count, other cytokine (IL-2, IL-4, IL-17A, IFN-γ, and TNF-α) levels, or severity scores (PRISM-III, PIM-3, pSOFA) (all p ≥ 0.05).

Among the 189 children with septic shock, 76 had positive culture results for body fluids, including blood, sputum, pus, urine, feces, pleural effusion, ascites, and cerebrospinal fluid. Among these patients, 27 (35.53%) were in the G+ group, and 49 (64.47%) were in the G- group. There were no statistically significant differences in the blood biomarkers (WBC count; PCT count; and CRP, lactate, LDH, IL-2, IL-4, IL-6, IL-10, IL-17A, IFN-γ, and TNF-α levels) or critical illness scores (PRISM-III, PIM-3, and pSOFA) between the G+ group and G- group (all p values ≥ 0.05) (**Supplementary Table 3**).

## Discussion

In clinical practice, biological indicators are frequently used for the early diagnosis and prognosis of sepsis and septic shock in children. Common biological indicators include lactate level, PLT count, CRP level, and PCT level, of which lactate level and PLT count were included in the Phoenix score (2). In recent years, cytokine levels have been used as biological indicators in for the early diagnosis and prognosis of sepsis and septic shock (19, 20). Cytokines have pro- and anti-inflammatory functions, and their imbalance can be dysregulating, which may lead to a cytokine storm due to severe infection (4, 5, 21). As the main anti-inflammatory and pro-inflammatory cytokines, IL-6 and IL-10 play important roles in septic shock (22, 23).

Studies on adult sepsis have shown that the level of IL-6 contributes to the diagnosis and prognosis of septic shock (6, 24, 25). One study demonstrated that an IL-6 concentration exceeding 77 pg/ml can facilitate the diagnosis of septic shock (14). However, the value of the IL-6 level for predicting septic shock mortality is limited (14, 26, 27). Studies on pediatric sepsis and septic shock have shown that the IL-6 level can be used for early diagnosis and prognosis evaluation (8, 28), although the correlation between IL-6 levels and illness severity is relatively weak (9). Smok B et al. reported that the IL-6 level had high sensitivity (82.9%) and high specificity (81.6%) for the diagnosis of sepsis in children at the critical concentration of 7.5 pg/ml (8). However, the diagnostic criteria for sepsis in their study were established in 2005 and are no longer used. In this study, the plasma IL-6 concentration predicted the 28-day mortality of children with septic shock, and the optimal threshold was 414.92 pg/ml. In addition, the IL-6 level was also an independent risk factor for 28-day mortality in children with septic shock. Recently, one study demonstrated that IL-6 levels were correlated with the age of pediatric patients with sepsis (<5 years vs ≥5 years) (29). Our study found that plasma IL-6 concentration predicts 28-day mortality in children with septic shock. In recent years, studies have attempted to use CRRT to destroy cytokines and improve the outcomes of sepsis patients. One systematic review and meta-analysis revealed that treatment with the oXiris filter during CRRT could reduce IL-6 levels and may be associated with lower 28-, 7-, and 14-day mortalities in sepsis patients (30). However, the quality of the studies included in this meta-analysis was low or very low, and further research is needed to verify the findings (30).

IL-10 is strongly correlated with the development of sepsis and septic shock in adults (31, 32). In adult patients with septic shock, the levels of IL-10 were significantly greater in nonsurvivors than in survivors (33). The IL-10 level also has high discriminatory ability and accuracy for the early diagnosis of sepsis in children (34). Zeng et al. reported that the IL-10 level can be used as a diagnostic biomarker for sepsis in children (35). Another study revealed that the IL-10 level can predict late-onset neonatal sepsis (36). However, few studies have investigated the prognosis of septic shock in children. This study revealed that the level of IL-10 was related to the outcomes of children with septic shock. When the concentration of IL-10 was greater than the optimal threshold of 29.66 pg/m, the 28-day mortality increased. Therefore, IL-10 levels can help clinicians assess the severity of illness in children. Elevated levels of IL-6 and IL-10 are associated with death in children with septic shock, but the role of cytokines is complex, and further high-quality randomized controlled trials are needed to explore whether clearing these cytokines can have therapeutic effects and improve prognosis.

The role of cytokine (IL-2, IL-4, IL-17A, TNF-α, and IFN-γ) levels in sepsis and septic shock in children has been poorly studied. In the context of sepsis, some studies have shown significantly increased levels of IL-17A and TNF-α in childhood sepsis-related deaths (9). This study revealed that the levels of IL-2, IL-4, IL-17A, TNF-α, and IFN-γ increased on average in nonsurvivors but that only TNF-α and IFN-γ could predict 28-day mortality.

Infection with different pathogens may lead to different levels of cytokine expression. In this study, the most common pathogenic bacteria in children with septic shock was *Staphylococcus*. Among all the children whose bacterial cultures were positive, the percentage of children with G- bacteria was greater than that of those with G+ bacteria, and the most common type of G- bacteria was *Klebsiella*. This finding is consistent with previous research results (37). Previous studies have shown that the levels of IL-6 and IL-10 are useful for the early identification of gram-negative and gram-positive bacteremia and that the value of combined indicators was higher (38). IL-6 and IL-10 levels can also be used to identify gram-negative bacterial sepsis, regardless of the site of infection (bloodstream or nonblood stream infection) (11). However, this study revealed that the levels of IL-6, IL-10, IL-2, IL-4, IL-17A, IFN-γ, and TNF-α did not significantly differ between the G+ and the G- group in children with positive fluid cultures (blood, sputum, pus, urine, feces, pleural effusion, ascites, and cerebrospinal fluid). In children with positive blood cultures, the levels of the cytokines IL-6, IL-10, IL-2, IL-4, and IL-17A were greater in the G- group than in the G+ group, and the levels of IL-6 and IL-10 were significantly different between the two groups. It can be inferred that the levels of plasma IL-6 and IL-10 may help broaden early empirical anti-infection regimens targeting septic shock caused by bloodstream infections of G+ or G- bacteria, although precision antibiotic therapy in septic shock relies on blood culture results.

This study evaluated the value of cytokine (IL-2, IL-4, IL-6, IL-10, IL-17A, IFN-γ, and TNF-α) levels in identifying bacterial gram types in children with septic shock. It may assist clinicians in antimicrobial therapy and prognosis assessment.

This study has several limitations. First, this study was limited by the inherent factors of its retrospective design. Second, this was a single-center study, and the sample size was relatively small. However, we included all eligible cases of septic shock after our center began cytokine testing in July 2019 in the electronic health database. Third, the 28-day mortality rate of children with septic shock may be related to underlying diseases, and cytokine levels may also be affected by comorbidities. Fourth, the dynamic changes in cytokine levels were not investigated. Fifth, the guidelines for pediatric sepsis [2,3] do not provide recommendations for cytokine testing. Some children with septic shock did not undergo cytokine testing within 24 hours of admission to PICU and were excluded, which may have some impact on the research results. Finally, a multicenter prospective study with a large sample is needed to investigate the differences in cytokine levels and profiles during septic shock caused by different pathogenic microorganisms.

## Conclusion

In children with septic shock, the levels of IL-6, IL-10, IFN-γ, and TNF-α were significantly increased in the nonsurvivor group than in the survivor group. The levels of IL-6, IL-10, IFN-γ, and TNF-α were able to predict 28-day mortality. IL-6 was an independent risk factor associated with 28-day mortality. Furthermore, IL-6 and IL-10 levels were significantly increased in bloodstream infections caused by gram-negative bacteria compared to bloodstream infections caused by gram-positive bacteria in children with septic shock. It is worth noting that IL-6 and IL-10 may not only be used as biological indicators of outcomes of children with septic shock but also may be helpful for guiding empirical antibiotic coverage.

## Data Availability

The raw data supporting the conclusions of this article will be made available by the authors, without undue reservation.

## Supplementary material

Supplementary Figure 1 ROC curve analysis for 28-day mortality in children with septic shock. Levels of IL-6, IL-10, IFN-γ, IL-6+IL-10, and IL-6+IFN-γ levels predict mortality, showing sensitivity versus 1-specificity. Curves represent IL-6 (AUC = 0.64), IL-10 (AUC = 0.68), IFN-γ (AUC = 0.64), IL-6 + IL-10 (AUC = 0.68), and IL-6 + IFN-γ (AUC = 0.68). Diagonal line indicates random performance.
