# Supplementary material for: Role of cytokine levels in pathogen classification and prognosis of pediatric septic shock
Source: Front Immunol. 2026 Jan 27;17:1714948. doi: 10.3389/fimmu.2026.1714948 (PMC12886044; doi:10.3389/fimmu.2026.1714948)
Supplement: Supplementary Figure 1 — ROC curve analysis for 28-day mortality in children with septic shock. Levels of IL-6, IL-10, IFN-γ, IL-6+IL-10, and IL-6+IFN-γ levels predict mortality, showing sensitivity versus 1-specificity. Curves represent IL-6 (AUC = 0.64), IL-10 (AUC = 0.68), IFN-γ (AUC = 0.64), IL-6 + IL-10 (AUC = 0.68), and IL-6 + IFN-γ (AUC = 0.68). Diagonal line indicates random performance. [file DataSheet1.pdf]

Supplementary Table 1 Characteristics of cytokine levels in children of different ages.

	< 5 years			≥ 5 years		
	**Survivors (n =58)**	**Nonsurvivors (n = 33)**	**p**	**Survivors (n = 63)**	**Nonsurvivors (n = 35)**	**p**
IL-2(pg/ml), M (IQR)	0.87 (0.01, 3.85)	0.84 (0.35, 1.62)	0.484	0.51 (0.01, 1.52)	0.83 (0.34, 1.76)	0.221
IL-4(pg/ml), M (IQR)	0.48 (0.01, 2.48)	0.48 (0.01, 1.56)	0.318	0.22 (0.00, 1.22)	0.87 (0.13, 1.34)	0.057
IL-6(pg/ml), M (IQR)	628.30 (54.51, 4165.40)	1867.50 (510.78, 14087.71)	0.045	204.85 (39.03, 2792.23)	3275.33 (156.86, 13048.52)	0.013
IL-10(pg/ml), M (IQR)	56.92 (13.53, 287.82)	108.25 (30.19, 871.76)	0.060	23.53 (5.95, 153.75)	262.62 (41.81, 964.31)	<0.001
IL-17A(pg/ml), M (IQR)	2.40 (0.01, 6.12)	3.72 (0.01, 6.45)	0.556	1.15 (0.01, 7.74)	4.06 (1.04, 7.96)	0.046
IFN-γ(pg/ml), M (IQR)	1.18 (0.07, 6.01)	2.99 (0.99, 39.93)	0.040	1.32 (0.34, 4.72)	4.32 (0.57, 18.61)	0.021
TNF-α(pg/ml), M (IQR)	1.30 (0.53, 4.56)	1.64 (0.97, 3.59)	0.496	0.70 (0.03, 1.77)	1.58 (0.74, 3.77)	0.007

IQR interquartile range; M, median; IFN-γ, interferon-γ; IL-2, interleukin-2; IL-4, interleukin-4; IL-6, interleukin-6; IL-10, interleukin-10; IL-17A, interleukin 17A; TNF-α, tumor necrosis factor-alpha.

Supplementary Table 2 Characteristics of cytokine levels in different illness severity.

Variables	pSOFA ≤ 5 Survivors (n = 55)	pSOFA ≤ 5 Nonsurvivors (n = 19)	p	pSOFA > 5 Survivors (n = 66)	pSOFA > 5 Nonsurvivors (n = 49)	p
IL-2 (pg/ml), M (IQR)	0.39 (0.01, 1.52)	0.52 (0.06, 0.99)	0.886	0.73 (0.01, 2.58)	0.98 (0.42, 1.81)	0.896
IL-4 (pg/ml), M (IQR)	0.45 (0.01, 1.21)	0.59 (0.03, 1.17)	0.632	0.32 (0.01, 1.85)	0.87 (0.01, 1.72)	0.682
IL-6 (pg/ml), M (IQR)	159.87 (31.02, 2661.29)	974.55 (90.84, 11206.11)	0.115	505.82 (93.57, 3879.32)	3117.56 (424.20, 14087.71)	0.016
IL-10 (pg/ml), M (IQR)	20.06 (5.73, 87.06)	48.98 (15.67, 718.07)	0.018	64.32 (14.28, 384.63)	210.78 (47.96, 963.00)	0.011
IL-17A (pg/ml), M (IQR)	0.01 (0.00, 5.48)	2.50 (0.01, 7.36)	0.21	2.95 (0.01, 8.20)	3.84 (1.79, 6.84)	0.573
IFN-γ (pg/ml), M (IQR)	1.02 (0.08, 2.31)	1.99 (0.48, 7.27)	0.083	1.48 (0.33, 6.43)	4.32 (0.99, 39.93)	0.027
TNF-α (pg/ml), M (IQR)	0.70 (0.01, 1.89)	1.10 (0.49, 2.12)	0.292	1.06 (0.41, 2.94)	1.65 (0.97, 3.84)	0.063

IFN-γ, interferon-γ; IL-2, interleukin-2; IL-4, interleukin-4; IL-6, interleukin-6; IL-10, interleukin-10; IL-17A, interleukin 17A; pSOFA, Pediatric Sequential Organ Failure Assessment; TNF-α, tumor necrosis factor-alpha.

Supplementary Table 3 Laboratory parameter results from all fluid culture results.

Variables	Total (n = 76)	G+ group (n = 27)	G- group (n = 49)	p
WBC(x10⁹/l), M (IQR)	5.13 (1.76, 12.14)	6.83 (2.08,14.44)	4.56 (1.20,10.18)	0.158
CRP(mg/l), M (IQR)	71.23 (19.79, 126.75)	62.96 (17.54,112.70)	76.00 (35.65,131.37)	0.641
PCT(ng/ml), M (IQR)	32.07 (5.13, 100.00)	36.94 (4.75,100.00)	26.06 (6.45,100.00)	0.796
Lactate(mmol/L), M (IQR)	2.30 (1.30, 3.60)	1.70 (1.15,3.10)	2.30 (1.50,3.60)	0.178
LDH(U/L), M (IQR)	433.15 (309.50, 797.42)	537.00 (340.50,812.95)	367.00 (289.00,706.00)	0.146
IL-2(pg/ml), M (IQR)	1.01 (0.27, 2.50)	1.27 (0.41,2.44)	0.99 (0.10,2.50)	0.704
IL-4(pg/ml), M (IQR)	0.60 (0.01, 1.40)	0.62 (0.01,1.23)	0.59 (0.03,1.88)	0.546
IL-6(pg/ml), M (IQR)	1825.66 (69.53, 10972.59)	510.78 (46.08,2781.47)	4199.09 (95.03,14537.18)	0.082
IL-10(pg/ml), M (IQR)	108.56 (16.70, 757.27)	64.65 (16.59,190.80)	235.82 (17.79,1205.71)	0.111
IL-17A(pg/ml), M (IQR)	2.72 (0.01, 7.41)	2.94 (0.01,8.04)	2.65 (0.01,7.32)	0.667
IFN-γ(pg/ml), M (IQR)	2.07 (0.47, 5.31)	2.50 (0.56,10.77)	1.61 (0.47,4.32)	0.323
TNF-α(pg/ml), M (IQR)	1.19 (0.22, 3.43)	1.24 (0.64,3.76)	1.15 (0.15,3.43)	0.497
PRISM-III, M (IQR)	13.00 (8.75, 17.00)	14.00 (5.00,17.00)	13.00 (10.00,16.00)	0.849
PIM-3, M (IQR)	0.02 (0.01, 0.04)	0.03 (0.01,0.04)	0.02 (0.01,0.04)	0.791
pSOFA,M (IQR)	6.50 (4.00, 9.00)	7.00 (4.00,9.00)	6.00 (4.00,9.00)	0.694

IQR interquartile range; M, median; CRP, C-reactive protein; G-, gram-negative; G+, gram-positive; IFN-γ, interferon-γ; IL-2, interleukin-2; IL-4, interleukin-4; IL-6, interleukin-6; IL-10, interleukin-10; IL-17A, interleukin 17A; LDH, lactate dehydrogenase; PCT, procalcitonin; PIM-3, International Society of Pediatric Index of Mortality-3; PRISM-III, Pediatric Risk of Mortality-III; pSOFA, Pediatric Sequential Organ Failure Assessment; TNF-α, tumor necrosis factor-alpha; WBC, white blood cell.

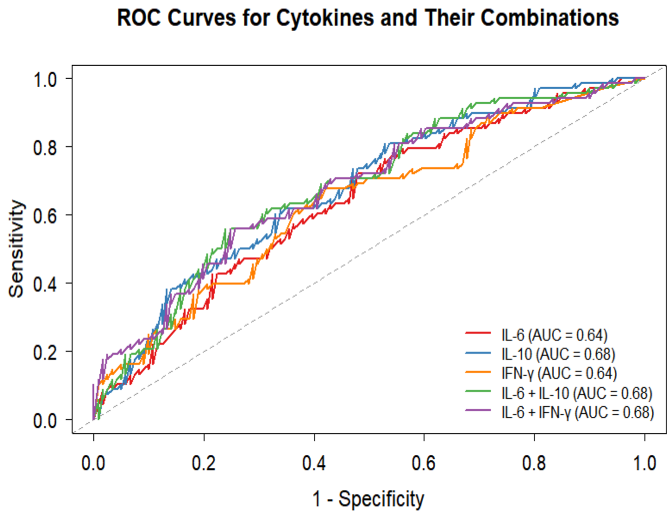

Supplementary FIGURE 1 ROC curve analysis for 28-day mortality in children with septic shock. Levels of IL-6, IL-10, IFN-γ, IL-6+IL-10, and IL-6+IFN-γ levels predict mortality, showing sensitivity versus 1-specificity. Curves represent IL-6 (AUC = 0.64), IL-10 (AUC = 0.68), IFN-γ (AUC = 0.64), IL-6 + IL-10 (AUC = 0.68), and IL-6 + IFN-γ (AUC = 0.68). Diagonal line indicates random performance.
